# The Use of Various Measurement Methods for Estimating the Fracture Energy of PLA (Polylactic Acid)

**DOI:** 10.3390/ma15238623

**Published:** 2022-12-02

**Authors:** Luyao Gao, Aleksey D. Drozdov

**Affiliations:** Department of Materials and Production, Aalborg University, Pontoppidanstræde 103, 9220 Aalborg, Denmark

**Keywords:** PLA, essential work of fracture, dynamic fracture, PLA toughness

## Abstract

The essential work of fracture (EWF) and Izod/Charpy impact tests have been used to investigate the fracture toughness in the plane stress of brittle polymers. In this paper, we had three goals: first, we aimed to employ how to estimate PLA toughness in different geometries; then, we proposed to compare Izod and Charpy Impact toughness in the same geometry; finally, we intended to determine the difference between EWF toughness and dynamic toughness. The results showed that the EWF method could be applied to evaluate PLA fracture behavior with small ligaments (2–4 mm), while the dynamic test could be employed with larger ligaments (5–7 mm). A comparison of the two impact test results obtained the following conclusions: Charpy impact toughness was higher than Izod impact toughness in the same geometry, and the impact toughness under a notch angle of 90° was larger than that of an angle of 45°. Both EWF and dynamic tests can be used to explore PLA toughness with small ligaments. The fracture energy decreases with ligament size in the EWF test, but it increases in the dynamic test.

## 1. Introduction

Polylactic acid (PLA) has been widely applied in many bio-related and medical-related fields due to its great advantages: biocompatibility, biodegradability, thermal stability, solvent resistance, gloss, and transparency; however, the disadvantage of low toughness limits PLA’s applications in a number of fields [1,2]. For this reason, many researchers have focused on approaches to increase the toughness of PLA such as plasticization, copolymerization, blending with other tough polymers, and adding elastomers [3]. Nonetheless, different opinions have emerged on the definitions and testing methods for toughness [4,5,6].

According to the literature, different methods of measuring toughness produce different results [7,8,9]. Therefore, we need to explore the differences between them. Many researchers have studied the toughness of polymers using the approach of the essential work of fracture (EWF). The EWF method can be used when a large plastic deformation exists during the fracture process, with numerous cavities occurring and cracks at the crack tip [10]. However, the parameters of EWF are affected by different experiment conditions and factors. The special energy of fracture is less related to the ligament length, especially when the ligaments are long, but it is influenced by the thickness of samples. The essential work of fracture is normally decreased due to the higher strain rate at the crack tip for higher temperature [11]. UV radiation can induce polymer chain break and decrease plastic deformation capacity, which result in obvious decline of the essential work of fracture [12]. In fracture behavior, crack initiation requires more energy than crack propagation [13]. The essential work of fracture in the yielding stage is greater than the tearing stage. Normally, the EWF test is operated with double-edge notched tensile (DENT) geometry. Nevertheless, it can sometimes be adopted in other geometries. For example, EWF methodology can be used in deeply double-edge notched small punch (DDEN-SP) test [14] and single-edge notched bending (SENB) as well [15]. EWF can be a simple alternative method for the J-integral under plane strain conditions.

Some research has used the Izod and Charpy impact methods to verify the fracture behavior of materials. The impact toughness is determined by the quantity, size, distribution, and the mechanical stability of partials [16]. In the Izod impact test, a fan-shape whitening zone appears in the crack tip, which is a spread of the shear-yielding region [17]. Crack propagation happens in the stress whitened zone while the crack grows, quickly just with low-energy absorption, in the brittle zone [18]. EWF theory can also be used in impact test when the fracture toughness is independent of sample thickness under a plane strain fracture condition.

In this paper, the fracture toughness of pure PLA was simultaneously investigated with three approaches (EWF test, Izod impact test, and Charpy impact test). Although there are many studies on the toughness of PLA, fewer researchers have focused on comparing different methods to measure toughness. In this study, we aimed to find the relationship between them.

## 2. Materials and Methods

### 2.1. Materials and Injection Moulding Conditions

In this work, the used material (PLA) is supplied by NatureWorks^®^ (3052D) and is specially designed for injection molding equipment. It has density of 1.24 g/cm^3^ and melt flow rate of 14 g/10 min (210 °C, 2.16 kg) according to ASTM D 1238.

Dumbbell-shaped specimens and prismatic bars (length (L) 70 mm, width (W) 10 mm, and thickness (t) 3 mm) were obtained by injection molding machine. The pellets were pre-dried at 60 °C for 24 h in the vacuum drying oven. The parameters for injection molding are shown in Table 1.

### 2.2. The Essential Work of Fracture (EWF)

**Figure 1:**
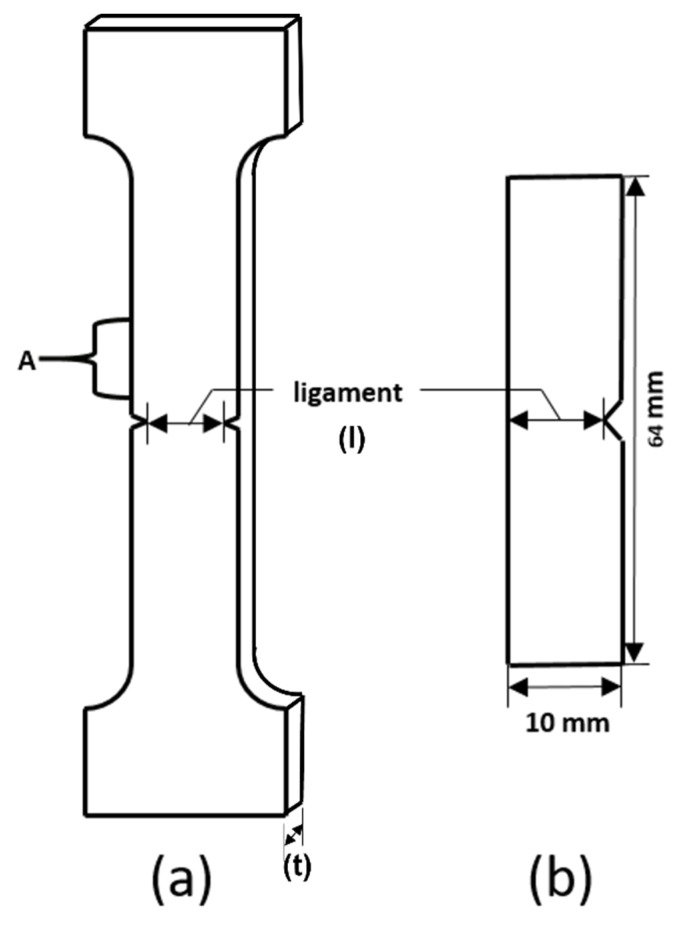
The geometry of samples for different toughness testing method: (**a**) EWF test, (**b**) dynamic test.

All specimens were pre-notched in the DENT geometry (V-shape), as shown in Figure 1a. The ligament lengths (*l*) were 2 mm, 2.5 mm, 3 mm, 3.5 mm, 4 mm, 4.5 mm, 5 mm, 5.5 mm, 6 mm, 6.5 mm, 7 mm, 7.5 mm, 8 mm, 8.5 mm, 9 mm, and 9.5 mm; each class contained 5–8 specimens. To obtain exact ligament lengths, microscope measurement was used.

EWF tests were performed by the universal tensile testing machine at room temperature with the strain rate of 100 mm/min. Force-displacement testing adopted the clip gauge (10 mm) fixed in region A in Figure 1a.

According to EWF theory, the total fracture energy (*W_f_*) can be calculated by the area under the force–displacement curve (*∫ FOx*),which is always divided into two parts. One is along the fracture line (*W_e_*), proportional to ligament area (*l·t*), which means that crack growth is fast with minimal energy absorption. The other is developed in a volume of material surrounding the crack (*W_p_*), proportional to the volume of the yielding zone (*l^2^·t*), which indicates that crack propagation is stable with large deformation and energy absorption capability. Thereby, *W_f_*, *W_e_*, and *W_p_* can be written as follows:

*W_f_* = *W_e_* + *W_p_* = ∫ *FOx* = *w_e_lt* + *βw_p_l^2^t* (1)

Normalizing Equation (1) by l t follows:

w_f_ = w_e_ + βw_p_l (2)

where *w_f_* is the special work of fracture, *w_e_* is the essential work of fracture, *β* is the shape factor, *w_p_* is the non-essential work per volume unit, *l* is the ligament length, and *t* is the thickness.

### 2.3. Impact Toughness

The samples were pre-notched in the SENT geometry (V-shape), as shown in Figure 1b. The ligament lengths (*l*) were 5 mm, 5.5 mm, 6 mm, 6.5 mm, 7 mm, 7.5 mm, 8 mm, 8.5 mm, 9 mm, 9.5 mm; for each size, there were at least 5 samples. The exact ligament lengths were also measured with a microscope.

Izod and Charpy tests were performed by the universal impact testing machine at a room temperature, with the initial angle of hammer as 150°.

Unlike EWF toughness, the impact toughness refers to the energy per unit cross-sectional area at the notch.

## 3. Results

### 3.1. Analysis of PLA EWF Toughness

**Figure 2:**
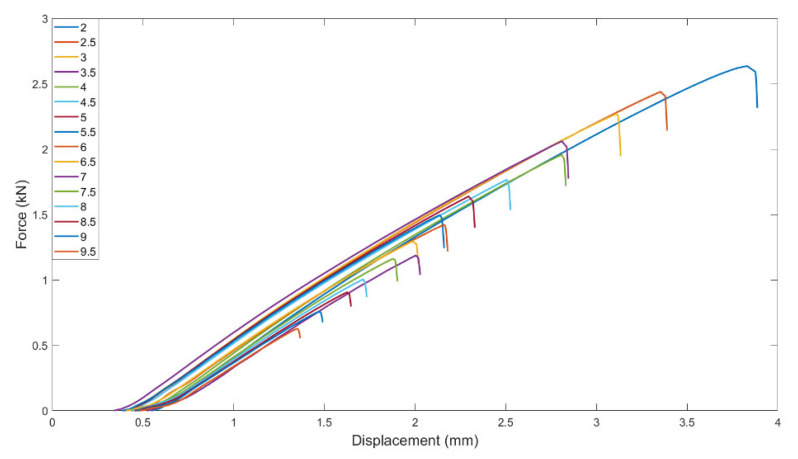
Force–displacement curves with different ligaments.

**Figure 3:**
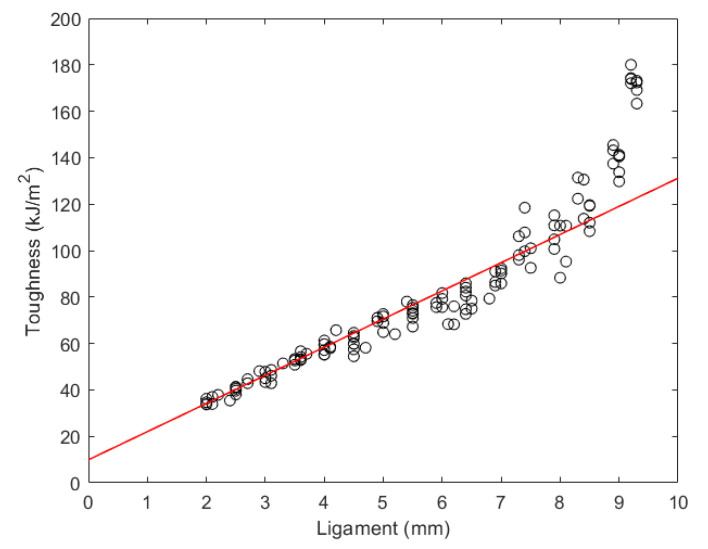
Special fracture toughness versus the ligament. Circles: experimental data on PLA samples. Solid line: Results of numerical simulation with $w_{0}=9.90\;$kJ/m^2^.

The force–displacement relationship with the ligaments from 2 mm to 9.5 mm was measured on the tension test machine. The results are shown in Figure 2. As observed, pure PLA exhibits an obviously brittle property. Toughness was calculated by the area under the force–displacement curve. Eventually, EWF toughness with various ligaments from 2 mm to 9.5 mm and the fitting curve from 2 mm to 4 mm were plotted in Figure 3. In Figure 3, toughness and ligament length demonstrate a visibly linear relationship in some ranges. It is known that measurements with the EWF method require two conditions:

(a) The ligament is under plane state of stress;
(b) The ligament is fully yielded prior to crack initiation and small enough to avoid edge effects.

The samples geometry must meet the following special condition to make sure the fracture is under plane stress [19].

(3) $$(3–5)\cdot t\leq l\;\leq \;\left(\frac{W}{3}\;or\;2r_{p}\right)$$

where *t* is the thickness, *l* is the ligament length, *W* is the width of specimen, and $r_{p}$ is the plastic zone size.

When the ligament was larger than 4 mm, it did not meet Equation (3); therefore, EWF measurements cannot be operated to analyze the samples with large ligaments.

A fitting curve indicates the relationship between toughness and ligament length. After calculating, the linear relationship is as follows:

*y* = 12.13 *x* + 9.90 (4)

The essential work of fracture toughness of pure PLA is 9.90 kJ/m^2^.

### 3.2. Dynamic Toughness

**Figure 4:**
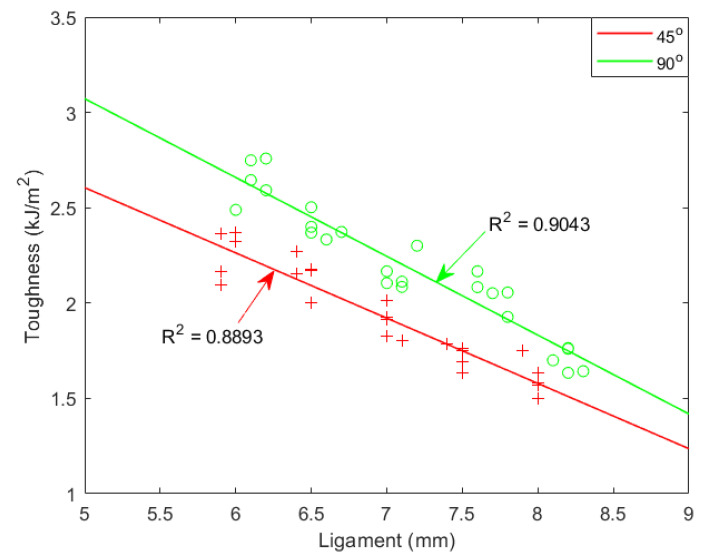
Izod impact toughness with various ligaments. ‘o’ represents Izod toughness with notch angle of 90°; ‘+’ represents Izod toughness with notch angle of 45°.

**Figure 5:**
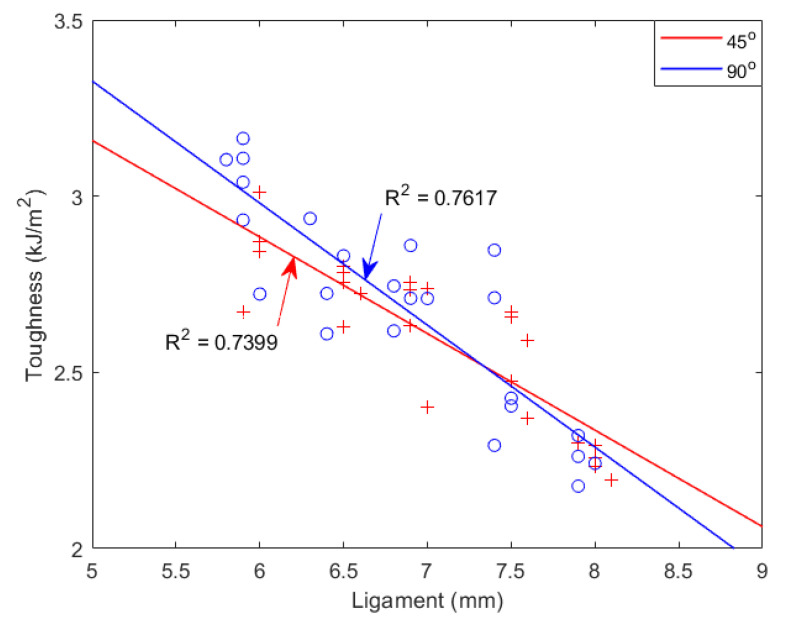
Charpy impact toughness with various ligaments. ‘o’ represents Izod toughness with notch angle of 90°; ‘+’ represents Izod toughness with notch angle of 45°.

To explore PLA dynamic toughness, we initially computed Izod and Charpy Impact toughness with the ligament length from 5 mm to 9.5 mm and plotted the results in Figure 4 and Figure 5, respectively. In these two figures, toughness and ligament length have a nonlinear relationship, whereas the impact process is not under plane stress over the full region. The impact test for a 5–7 mm ligament shows linear relationship but with a negative slope, because similar impact energy is absorbed in the outer plastic zone (OPZ) independent of ligament length and negligible work is absorbed in the fracture process zone (FPZ).

**Figure 6:**
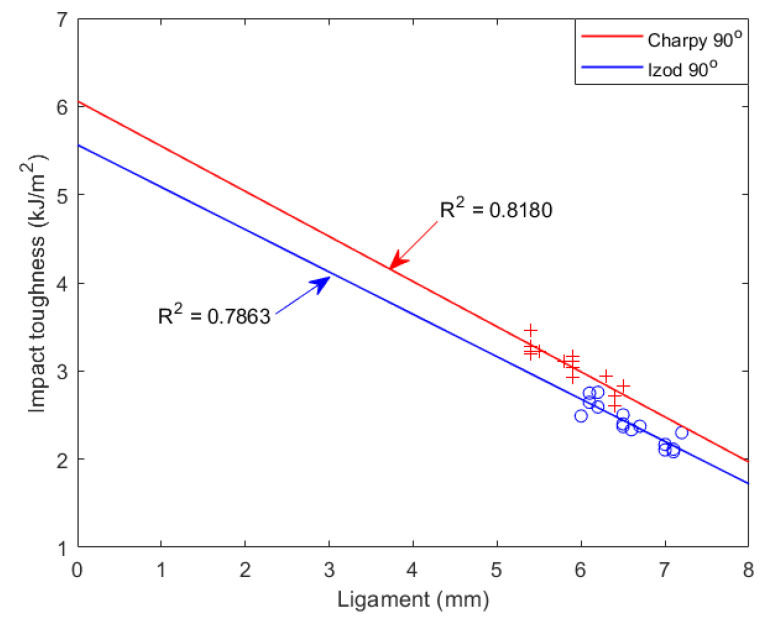
Dynamic toughness with various ligaments. ‘+’ represents Charpy toughness with notch angle of 90°; ‘o’ represents Izod toughness with notch angle of 90°. Results of numerical simulation with $w_{0}=5.56\;$and 6.06 kJ/m^2^, respectively.

From the dynamic experimental data, the fracture energy from 5–7 mm has a visible linear relationship. The fitting curves for Izod and Charpy 90° are presented in Figure 6. 

### 3.3. Diversity of EWF, Izod and Charpy Impact Tests

**Figure 7:**
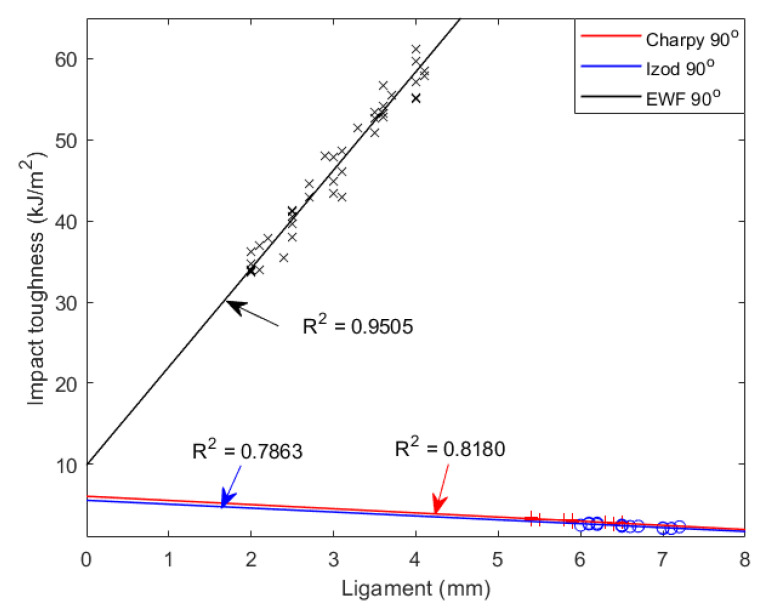
Comparison of the fracture energy calculated by different methods.

Fracture energy obtained from the EWF test and dynamic tests are listed in Table 2. The discrepancy between them are shown in Figure 7. DENT samples have larger outer plastic zone than SENT samples, which results in an outgoing toughness performance through EWF method. For EWF test, the valid fracture value belongs to the interval between 2–4 mm. At smaller ligament, the tensile test could not be operated. EWF fracture energy is 9.90 kJ/m^2^. For dynamic test, fracture values were gained from 5–7 mm. Izod fracture energy is 5.56 kJ/m^2^ and Charpy fracture energy is 6.06 kJ/m^2^.

## 4. Conclusions

In this article, we applied EWF and dynamic tests to measure pure PLA toughness. When the ligament was large, both methods failed to estimate the special fracture energy. For EWF test, the fracture toughness was 9.90 kJ/m^2^ with 2–4 mm ligaments. For the dynamic tests, the fracture toughness is 5.56 and 6.06 kJ/m^2^ with 5–7 mm ligaments. Comparing the two kinds of dynamic tests, Charpy impact toughness is higher than Izod. In the same impact test, samples with 90° notch are tougher than 45°.

Although the values for the EWF and dynamic tests are different, they can both express toughness property of PLA. From the results above, EWF toughness is 9.90 kJ/m^2^ and dynamic toughness is around 6 kJ/m^2^; thus, we predict the special fracture energy of pure PLA is in the range between 5–10 kJ/m^2^. The advantage of this approach is that if we could conduct tests with smaller ligaments, we reduce the uncertainty. The fracture toughness in dynamic tests increase with ligament length, while it decreases in EWF test.

## Figures and Tables

**Table 1:** The parameters for injection molding for PLA.

Parameters	Setting
Feed Zone (°C)	165
Transition Zone (°C)	185
Metering Zone (°C)	190
Mold Temperature (°C)	30
Screw Speed (rpm)	100–175
Holding Pressure (MPa)	80
Back Pressure (MPa)	0.35–0.69
Cooling Time (s)	17
Injection Speed (cm^3^/s)	20

**Table 2:** PLA toughness using three methods.

Method	Dimension (mm^3^)	Ligament (mm)	Notch Shape	Notch Angle (°)	Toughness (kJ/m^2^)
EWF	100 × 10 × 3	2–4	V	90	9.90
Charpy	64 × 3 × 10	5–7	V	90	6.06
Izod	64 × 3 × 10	5–7	V	90	5.56

## Data Availability

Not applicable.
